# Detection of SARS‐CoV‐2 B.1.617.2 (Delta) variant in three cats owned by a confirmed COVID‐19 patient in Harbin, China

**DOI:** 10.1002/vms3.715

**Published:** 2021-12-27

**Authors:** Kai Kang, Qi Chen, Yang Gao, Kai‐jiang Yu

**Affiliations:** ^1^ Department of Critical Care Medicine The First Affiliated Hospital of Harbin Medical University Harbin China; ^2^ Department of Critical Care Medicine Harbin Medical University Cancer Hospital Harbin China; ^3^ Department of Critical Care Medicine The Sixth Affiliated Hospital of Harbin Medical University Harbin China; ^4^ The Sino Russian Medical Research Center of Harbin Medical University Institute of Critical Care Medicine Harbin China

Dear Editor,

Heilongjiang Province is experiencing the fourth outbreak of coronavirus disease 2019 (COVID‐19) this year since a sudden case of severe acute respiratory syndrome coronavirus type 2 (SARS‐CoV‐2) infection was reported in the Second People's Hospital of Bayan County on 21 September 2021. The source of transmission is still unknown. Based on the SARS‐CoV‐2 genome sequence analysis results, the pathogenic source of this COVID‐19 outbreak was identified to be SARS‐CoV‐2 B.1.617.2 (Delta) variant. With the epidemic spread, three cats owned by a confirmed COVID‐19 patient were diagnosed with SARS‐CoV‐2 infection after two consecutive SARS‐CoV‐2 nucleic acid‐positive anal swab specimens. To our knowledge, this is the first report worldwide to show that cats can be definitely infected with SARS‐CoV‐2 Delta variant. In compliance with the regulations of the People's Republic of China on the Prevention and Control of Infectious Diseases, to avoid the spread of epidemic, these infected three cats were euthanised on 28 September.

SARS‐CoV‐2 is a highly pathogenic zoonotic pathogen mainly spread via human‐to‐human transmission. However, the infected population is by no means limited to humans. Bats were considered to be the original natural hosts and the most probable source of SARS‐CoV‐2 infection (Zhou et al., 2020). In addition, cats, dogs, ferrets and even tigers and lions have been repeatedly reported to be infected with SARS‐CoV‐2, but no specific case of SARS‐CoV‐2 Delta variant infection has been reported so far (Hamer et al., 2021; Sailleau et al., 2020). These stray or companion animals may be a silent intermediate host of SARS‐CoV‐2 and, therefore, should be screened during the outbreak of epidemic to avoid potential risk of human–animal–human transmission (Halfmann et al., 2020; Wang et al., 2020). Despite the lack of direct evidence of SARS‐CoV‐2 transmission from companion animals to humans, the contamination of the environment caused by infected companion animals, especially of closed environments, is of great concern as it is one of the pathways of SARS‐CoV‐2 transmission. The implications of these stray or companion animals, especially cats, dogs and other pets, as domestic SARS‐CoV‐2 hosts involved in disease dissemination require further research. In the meantime, family members and others in close contact with such animals, including veterinarians, should be considered at high risk of exposure.

In Chinese households, cats and dogs are the most common pets and are considered important family members by their owners. In contrast to humans, cats infected with SARS‐CoV‐2 rarely have fatal clinical manifestations and symptoms, except for mild to moderate respiratory and/or gastrointestinal symptoms (Hobbs & Reid, 2021). Despite the lack of effective targeted therapies and clinical experience, inspection and quarantine, isolation and appropriate medical treatment are feasible measures to be implemented in SARS‐CoV‐2‐infected pets. An urgent need exists to formulate relevant guidelines for the standardisation of the management of animals infected with SARS‐CoV‐2, the prevention of blind spots in epidemic prevention and control and the occurrence of death. The proper management of SARS‐CoV‐2‐infected pets does not mean abandoning them irresponsibly or compromising their welfare. Until more solid evidence of domestic animals acting as a source of spread, extreme actions such as euthanasia are unsupported and fearmongering may compromise animal welfare. Additional high‐quality research is necessary to improve the existing understanding of the transmission of COVID‐19 among the environment, humans and stray or companion animals, which would contribute to the formulation and implementation of effective targeted epidemic prevention and control policies in the future.

### PEER REVIEW

The peer review history for this article is available at https://publons.com/publon/10.1002/vms3.715.

## ETHICS STATEMENT

The authors confirm that the ethical policies of the journal have been adhered to.

## FUNDING

The National Natural Science Foundation of China, No.81902000 and 81770276.

## CONFLICT OF INTEREST

The authors declare that there are no conflicts of interests.

## AUTHOR CONTRIBUTIONS

Kai Kang: writing – review & editing. Qi Chen: writing – review & editing. Yang Gao: conceptualisation; formal analysis and funding acquisition.Kai‐jiang Yu:conceptualisation.

## Data Availability

The letter describes the cat euthanasia event derived from Chinese public news information. The data that support the findings of this study are available in PubMed.
